# Supplementary material for: A Humanized Yeast Phenomic Model of Deoxycytidine Kinase to Predict Genetic Buffering of Nucleoside Analog Cytotoxicity
Source: Genes (Basel). 2019 Sep 30;10(10):770. doi: 10.3390/genes10100770 (PMC6826991; doi:10.3390/genes10100770)
Supplement: Supplementary file 1 [file genes-10-00770-s001.zip › Additional_Files/Additional_File7_GOTermHeatmaps.zip!/Additional_File7_GOTermHeatmaps/RNA_splicing.pdf]

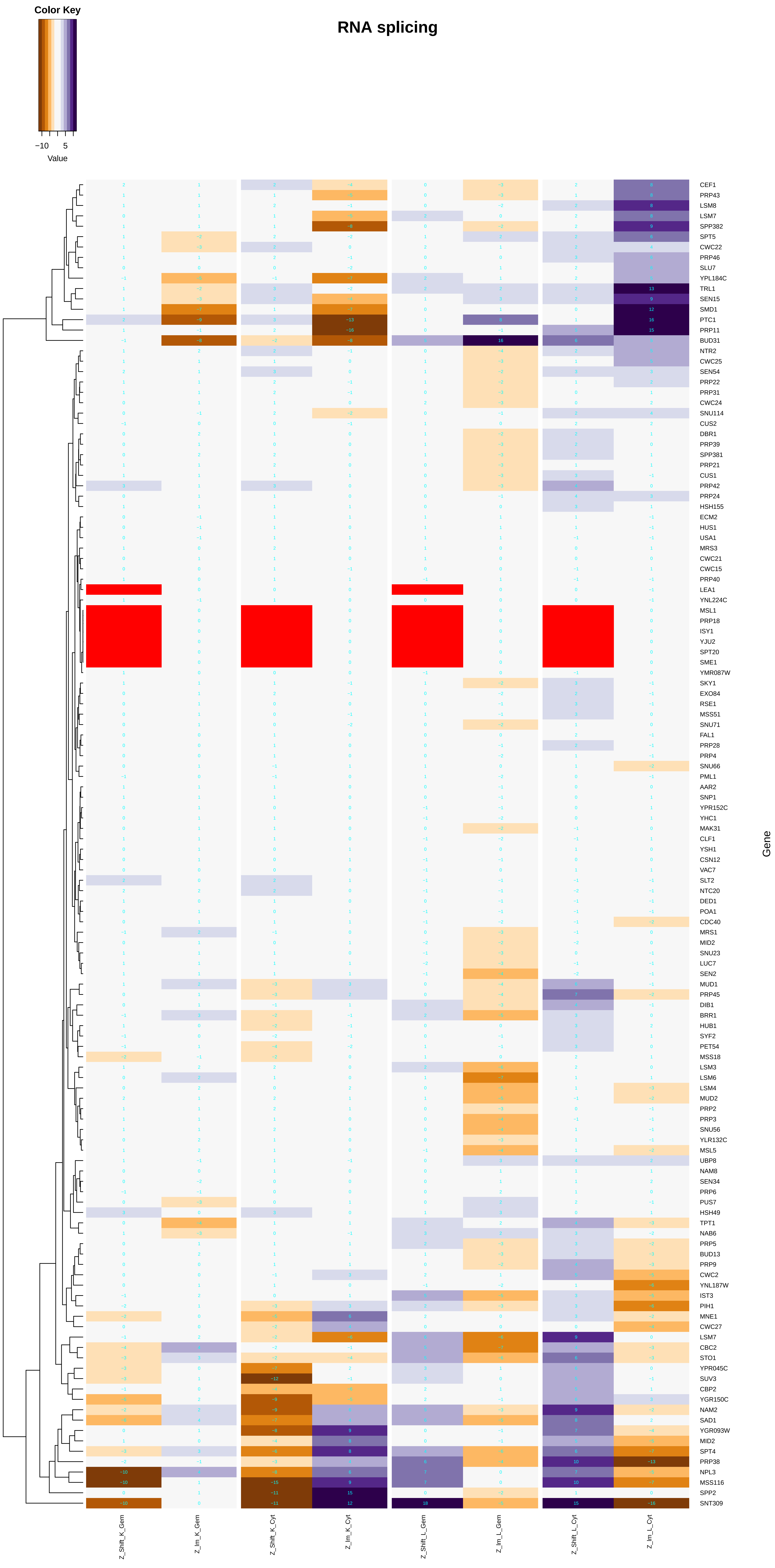
RNA splicing
Color Key
−10
5
Value
CEF1
PRP43
LSM8
LSM7
SPP382
SPT5
CWC22
PRP46
SLU7
YPL184C
TRL1
SEN15
SMD1
PTC1
PRP11
BUD31
NTR2
CWC25
SEN54
PRP22
PRP31
CWC24
SNU114
CUS2
DBR1
PRP39
SPP381
PRP21
CUS1
PRP42
PRP24
HSH155
ECM2
HUS1
USA1
MRS3
CWC21
CWC15
PRP40
LEA1
YNL224C
MSL1
PRP18
ISY1
YJU2
SPT20
SME1
YMR087W
SKY1
EXO84
RSE1
MSS51
SNU71
FAL1
PRP28
PRP4
SNU66
PML1
AAR2
SNP1
YPR152C
YHC1
MAK31
CLF1
YSH1
CSN12
VAC7
SLT2
NTC20
DED1
POA1
CDC40
MRS1
MID2
SNU23
LUC7
SEN2
MUD1
PRP45
DIB1
BRR1
HUB1
SYF2
PET54
MSS18
LSM3
LSM6
LSM4
MUD2
PRP2
PRP3
SNU56
YLR132C
MSL5
UBP8
NAM8
SEN34
PRP6
PUS7
HSH49
TPT1
NAB6
PRP5
BUD13
PRP9
CWC2
YNL187W
IST3
PIH1
MNE1
CWC27
LSM7
CBC2
STO1
YPR045C
SUV3
CBP2
YGR150C
NAM2
SAD1
YGR093W
MID2
SPT4
PRP38
NPL3
MSS116
SPP2
SNT309
Gene
Z_Shift_K_Gem
Z_Im_K_Gem
Z_Shift_K_Cyt
Z_Im_K_Cyt
Z_Shift_L_Gem
Z_Im_L_Gem
Z_Shift_L_Cyt
Z_Im_L_Cyt

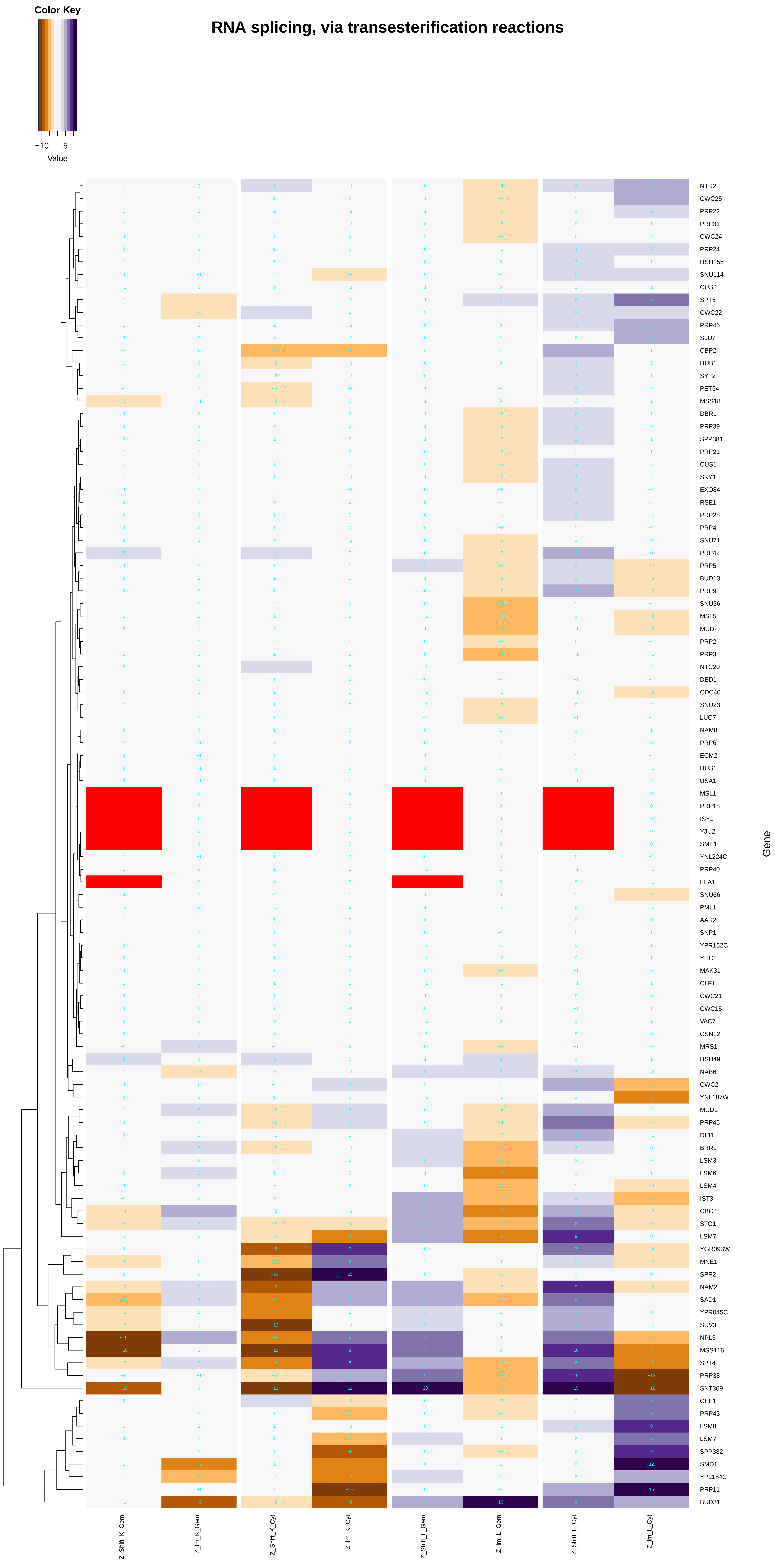
RNA splicing, via transesterification reactions
Color Key
−10
5
Value
NTR2
CWC25
PRP22
PRP31
CWC24
PRP24
HSH155
SNU114
CUS2
SPT5
CWC22
PRP46
SLU7
CBP2
HUB1
SYF2
PET54
MSS18
DBR1
PRP39
SPP381
PRP21
CUS1
SKY1
EXO84
RSE1
PRP28
PRP4
SNU71
PRP42
PRP5
BUD13
PRP9
SNU56
MSL5
MUD2
PRP2
PRP3
NTC20
DED1
CDC40
SNU23
LUC7
NAM8
PRP6
ECM2
HUS1
USA1
MSL1
PRP18
ISY1
YJU2
SME1
YNL224C
PRP40
LEA1
SNU66
PML1
AAR2
SNP1
YPR152C
YHC1
MAK31
CLF1
CWC21
CWC15
VAC7
CSN12
MRS1
HSH49
NAB6
CWC2
YNL187W
MUD1
PRP45
DIB1
BRR1
LSM3
LSM6
LSM4
IST3
CBC2
STO1
LSM7
YGR093W
MNE1
SPP2
NAM2
SAD1
YPR045C
SUV3
NPL3
MSS116
SPT4
PRP38
SNT309
CEF1
PRP43
LSM8
LSM7
SPP382
SMD1
YPL184C
PRP11
BUD31
Gene
Z_Shift_K_Gem
Z_Im_K_Gem
Z_Shift_K_Cyt
Z_Im_K_Cyt
Z_Shift_L_Gem
Z_Im_L_Gem
Z_Shift_L_Cyt
Z_Im_L_Cyt

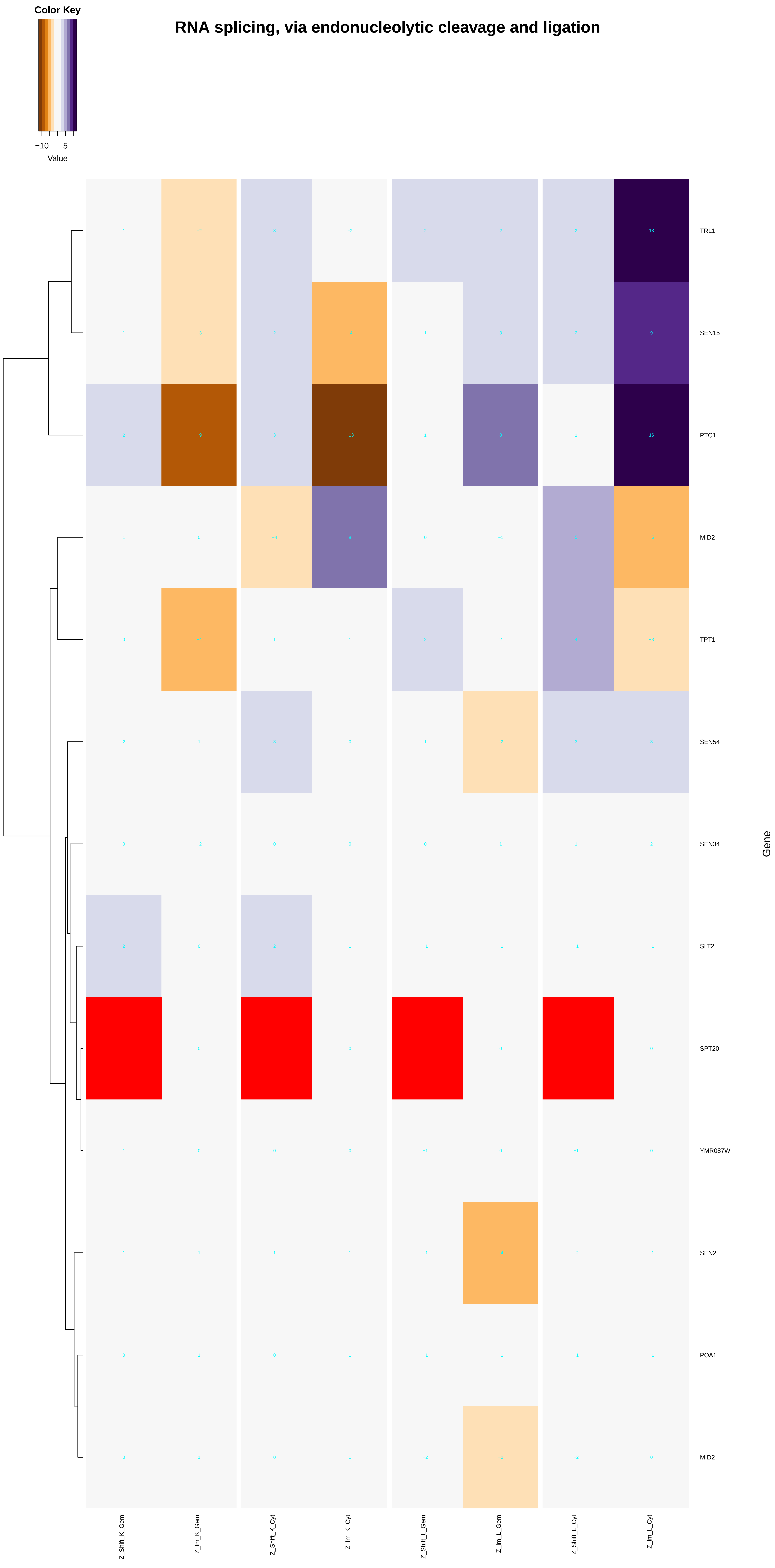

Color Key
−10 5
Value
RNA splicing, via endonucleolytic cleavage and ligation
TRL1
SEN15
PTC1
MID2
TPT1
SEN54
SEN34
SLT2
SPT20
YMR087W
SEN2
POA1
MID2
Gene
Z_Shift_K_Gem
Z_Im_K_Gem
Z_Shift_K_Cyt
Z_Im_K_Cyt
Z_Shift_L_Gem
Z_Im_L_Gem
Z_Shift_L_Cyt
Z_Im_L_Cyt

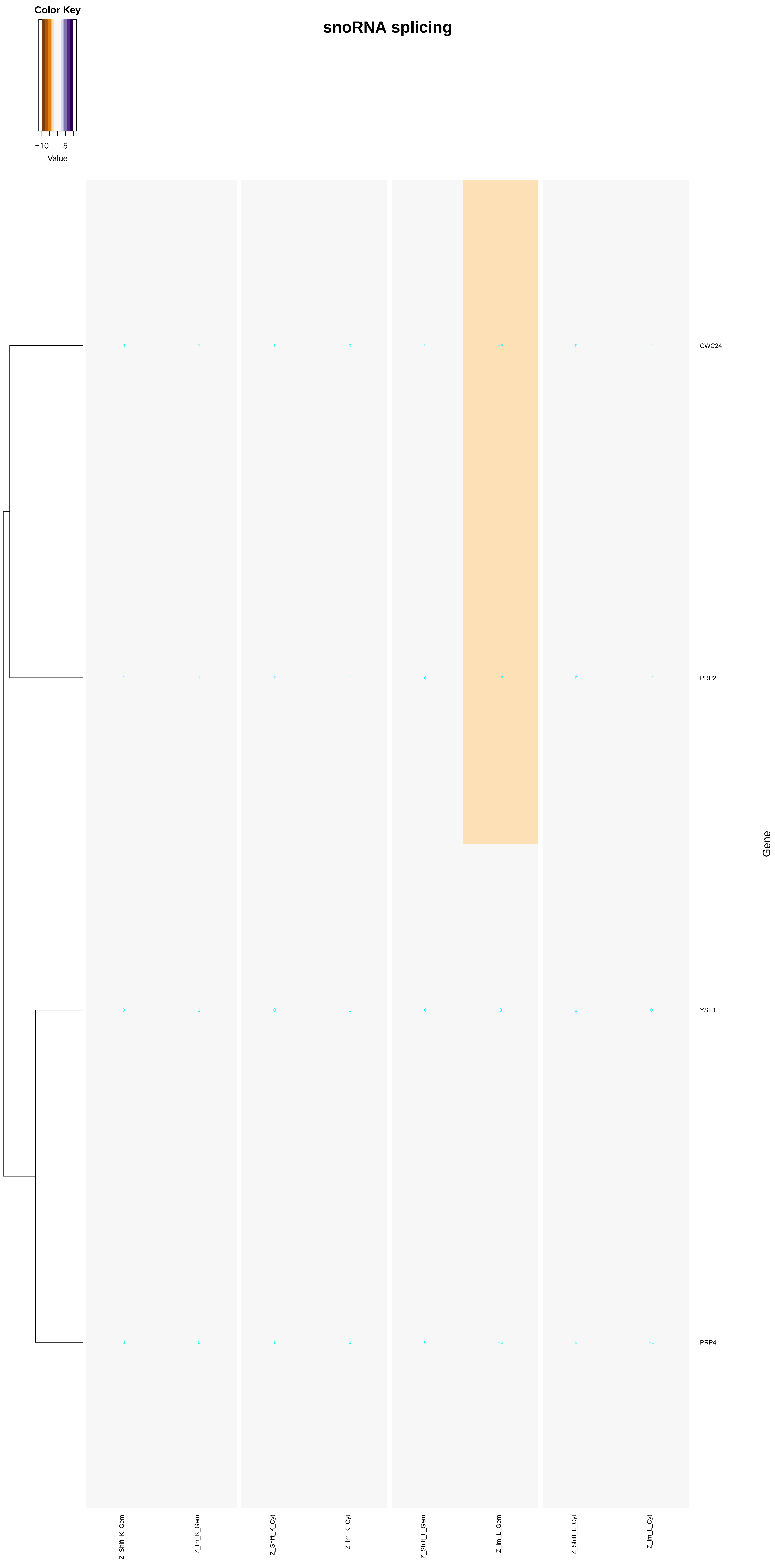
snoRNA splicing
Color Key
−10
5
Value
CWC24
PRP2
YSH1
PRP4
Gene
Z_Shift_K_Gem
Z_Im_K_Gem
Z_Shift_K_Cyt
Z_Im_K_Cyt
Z_Shift_L_Gem
Z_Im_L_Gem
Z_Shift_L_Cyt
Z_Im_L_Cyt

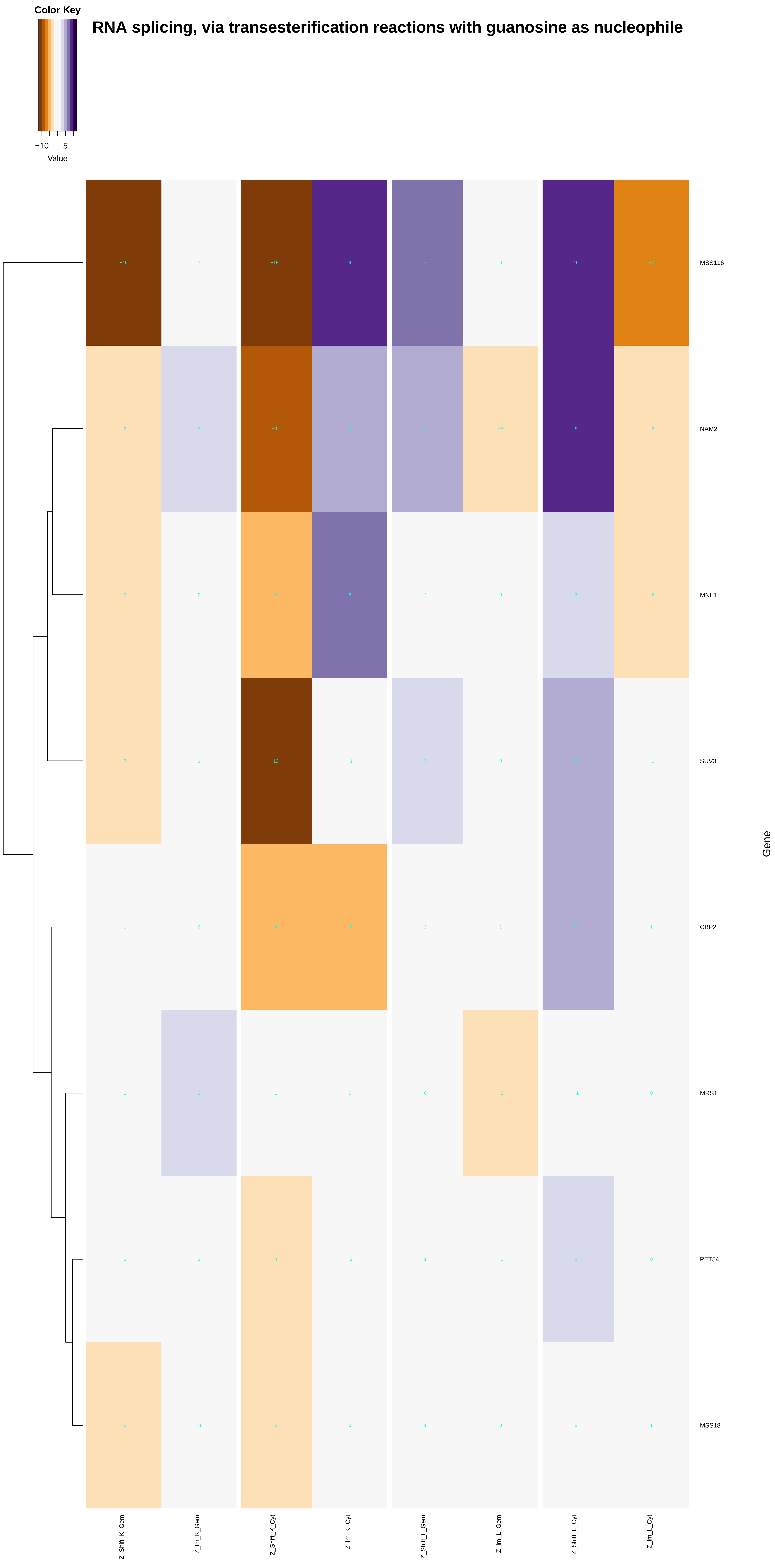
Color Key
RNA splicing, via transesterification reactions with guanosine as nucleophile
−10
5
Value
MSS116
NAM2
MNE1
SUV3
CBP2
MRS1
PET54
MSS18
Gene
Z_Shift_K_Gem
Z_Im_K_Gem
Z_Shift_K_Cyt
Z_Im_K_Cyt
Z_Shift_L_Gem
Z_Im_L_Gem
Z_Shift_L_Cyt
Z_Im_L_Cyt

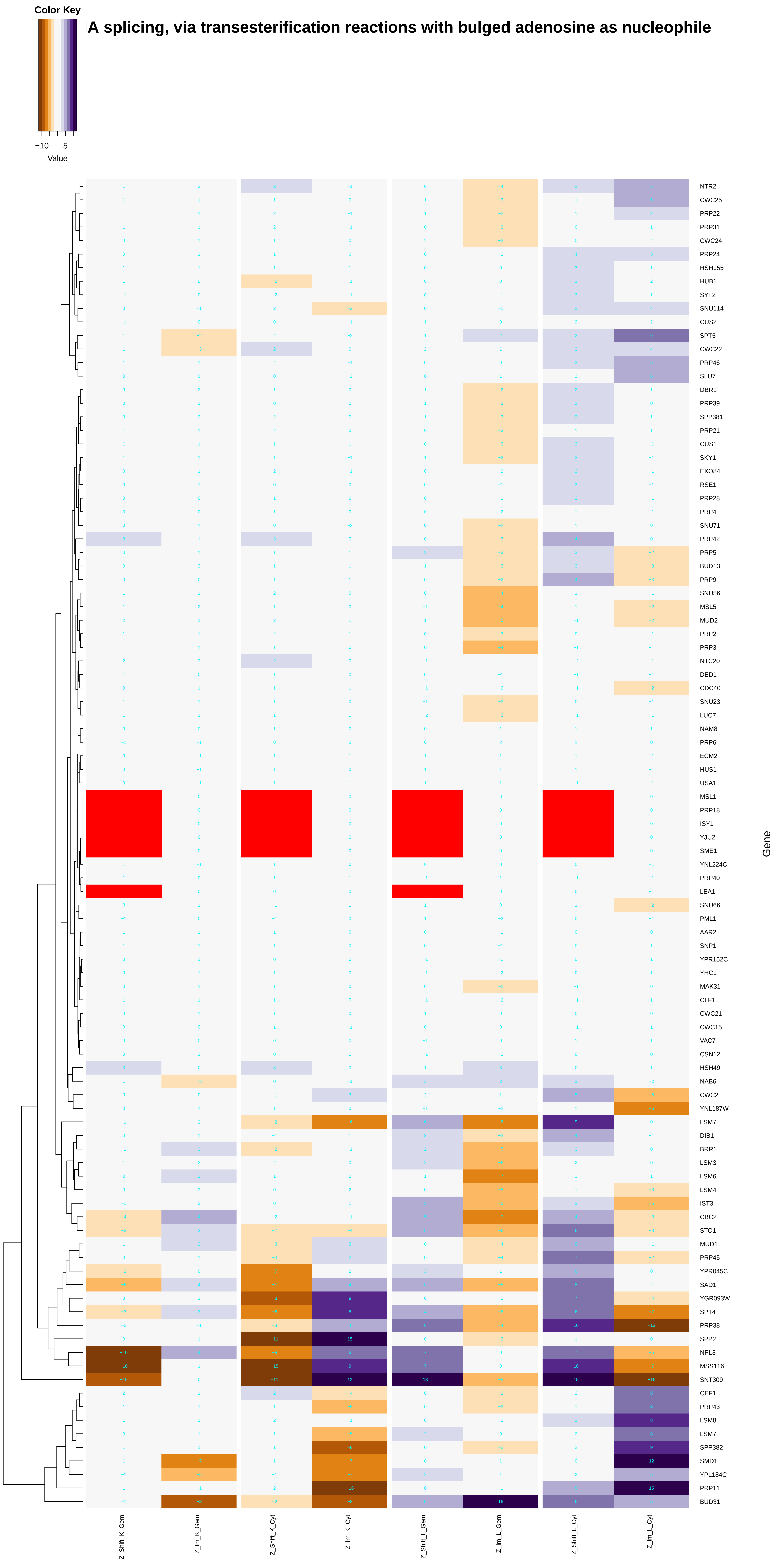

Color Key
−10
5
Value
A splicing, via transesterification reactions with bulged adenosine as nucleophile
NTR2
CWC25
PRP22
PRP31
CWC24
PRP24
HSH155
HUB1
SYF2
SNU114
CUS2
SPT5
CWC22
PRP46
SLU7
DBR1
PRP39
SPP381
PRP21
CUS1
SKY1
EXO84
RSE1
PRP28
PRP4
SNU71
PRP42
PRP5
BUD13
PRP9
SNU56
MSL5
MUD2
PRP2
PRP3
NTC20
DED1
CDC40
SNU23
LUC7
NAM8
PRP6
ECM2
HUS1
USA1
MSL1
PRP18
ISY1
YJU2
SME1
YNL224C
PRP40
LEA1
SNU66
PML1
AAR2
SNP1
YPR152C
YHC1
MAK31
CLF1
CWC21
CWC15
VAC7
CSN12
HSH49
NAB6
CWC2
YNL187W
LSM7
DIB1
BRR1
LSM3
LSM6
LSM4
IST3
CBC2
STO1
MUD1
PRP45
YPR045C
SAD1
YGR093W
SPT4
PRP38
SPP2
NPL3
MSS116
SNT309
CEF1
PRP43
LSM8
LSM7
SPP382
SMD1
YPL184C
PRP11
BUD31
Gene
Z_Shift_K_Germ
Z_lm_K_Germ
Z_Shift_K_Cyt
Z_lm_K_Cyt
Z_Shift_L_Germ
Z_lm_L_Germ
Z_Shift_L_Cyt
Z_lm_L_Cyt

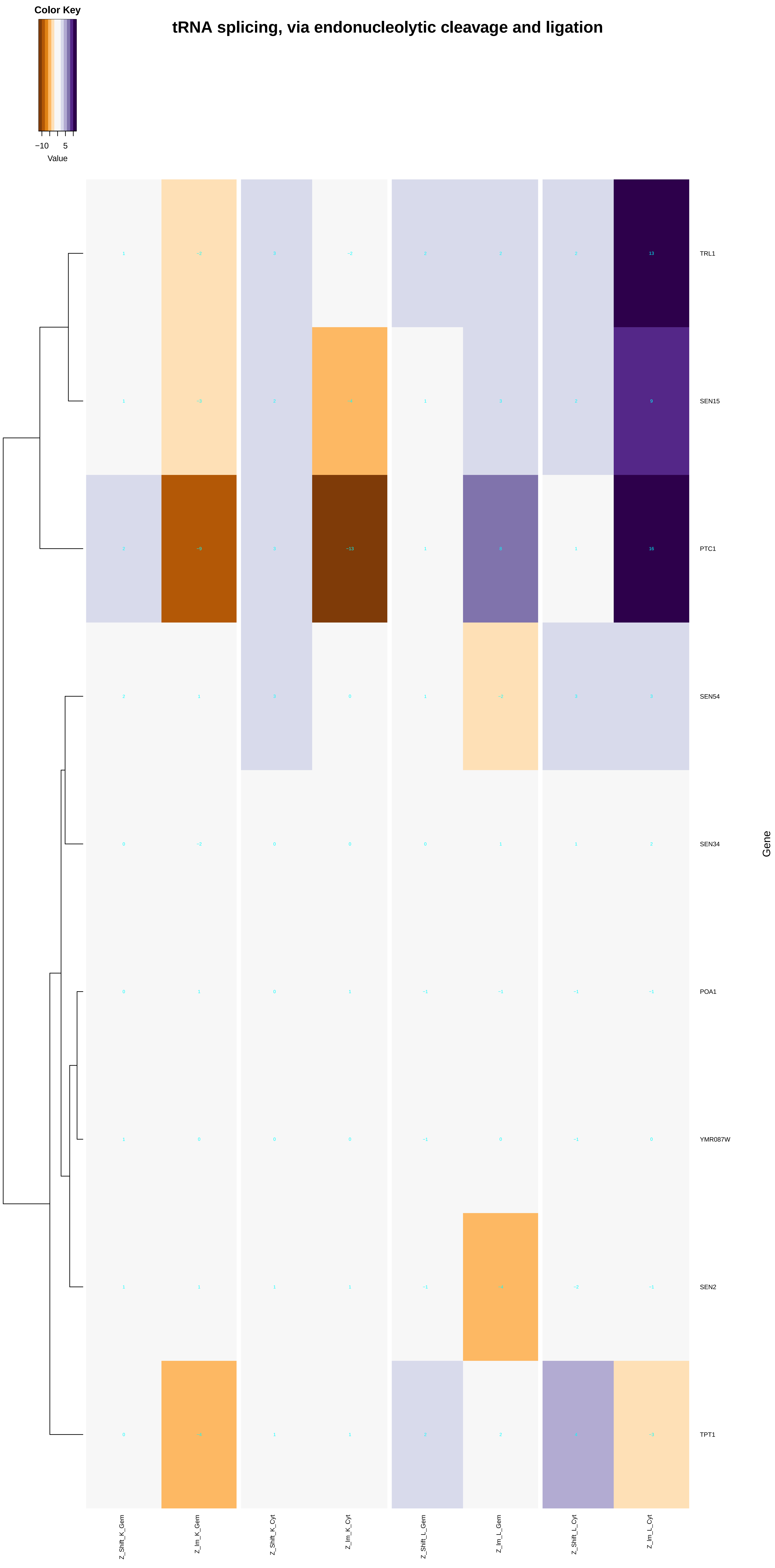
Color Key
−10
5
Value
tRNA splicing, via endonucleolytic cleavage and ligation
TRL1
SEN15
PTC1
SEN54
SEN34
POA1
YMR087W
SEN2
TPT1
Gene
Z_Shift_K_Gem
Z_Im_K_Gem
Z_Shift_K_Cyt
Z_Im_K_Cyt
Z_Shift_L_Gem
Z_Im_L_Gem
Z_Shift_L_Cyt
Z_Im_L_Cyt

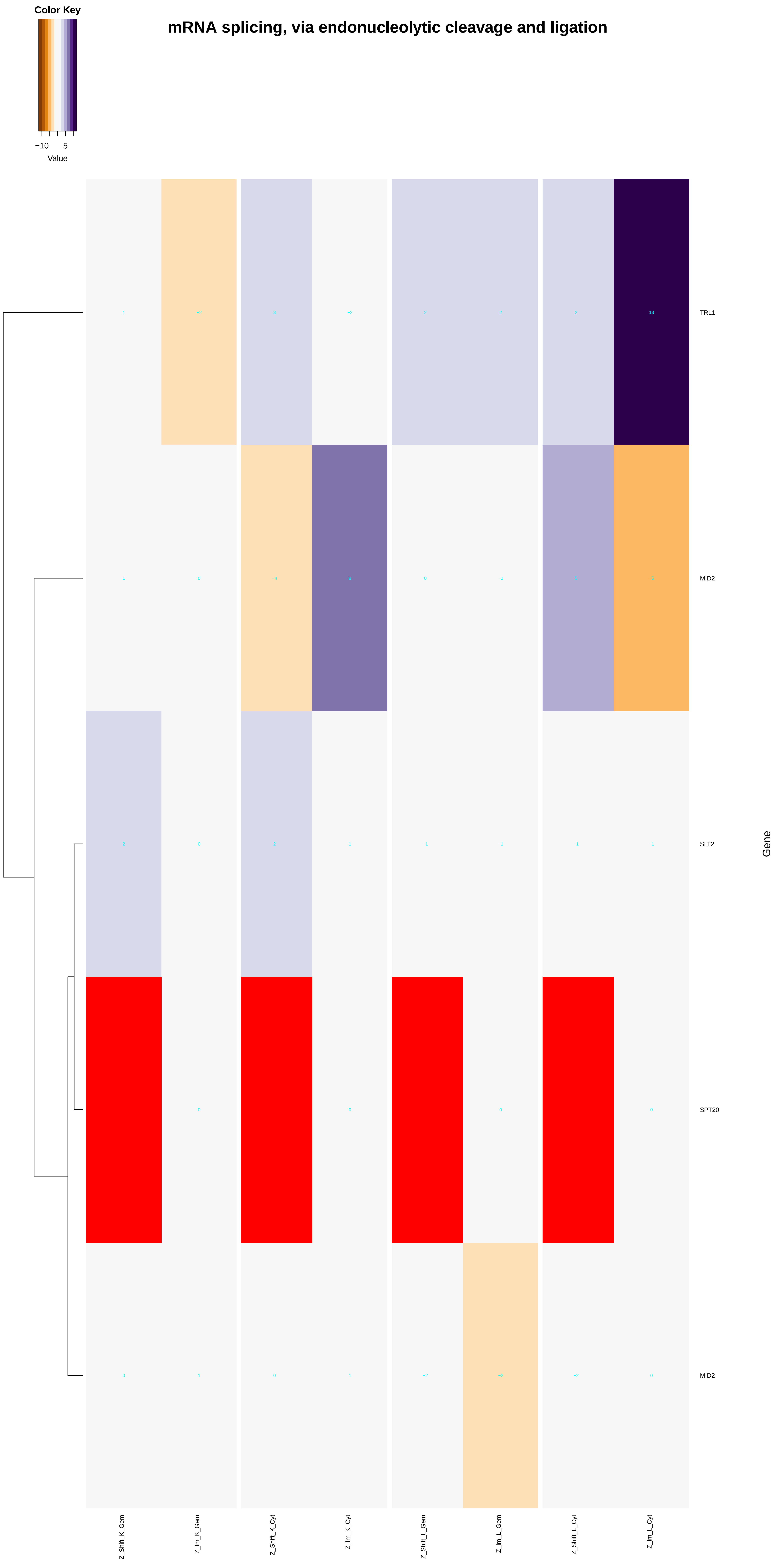

Color Key
−10
5
Value
mRNA splicing, via endonucleolytic cleavage and ligation
TRL1
MID2
SLT2
SPT20
MID2
Gene
Z_Shift_K_Gem
Z_Im_K_Gem
Z_Shift_K_Cyt
Z_Im_K_Cyt
Z_Shift_L_Gem
Z_Im_L_Gem
Z_Shift_L_Cyt
Z_Im_L_Cyt

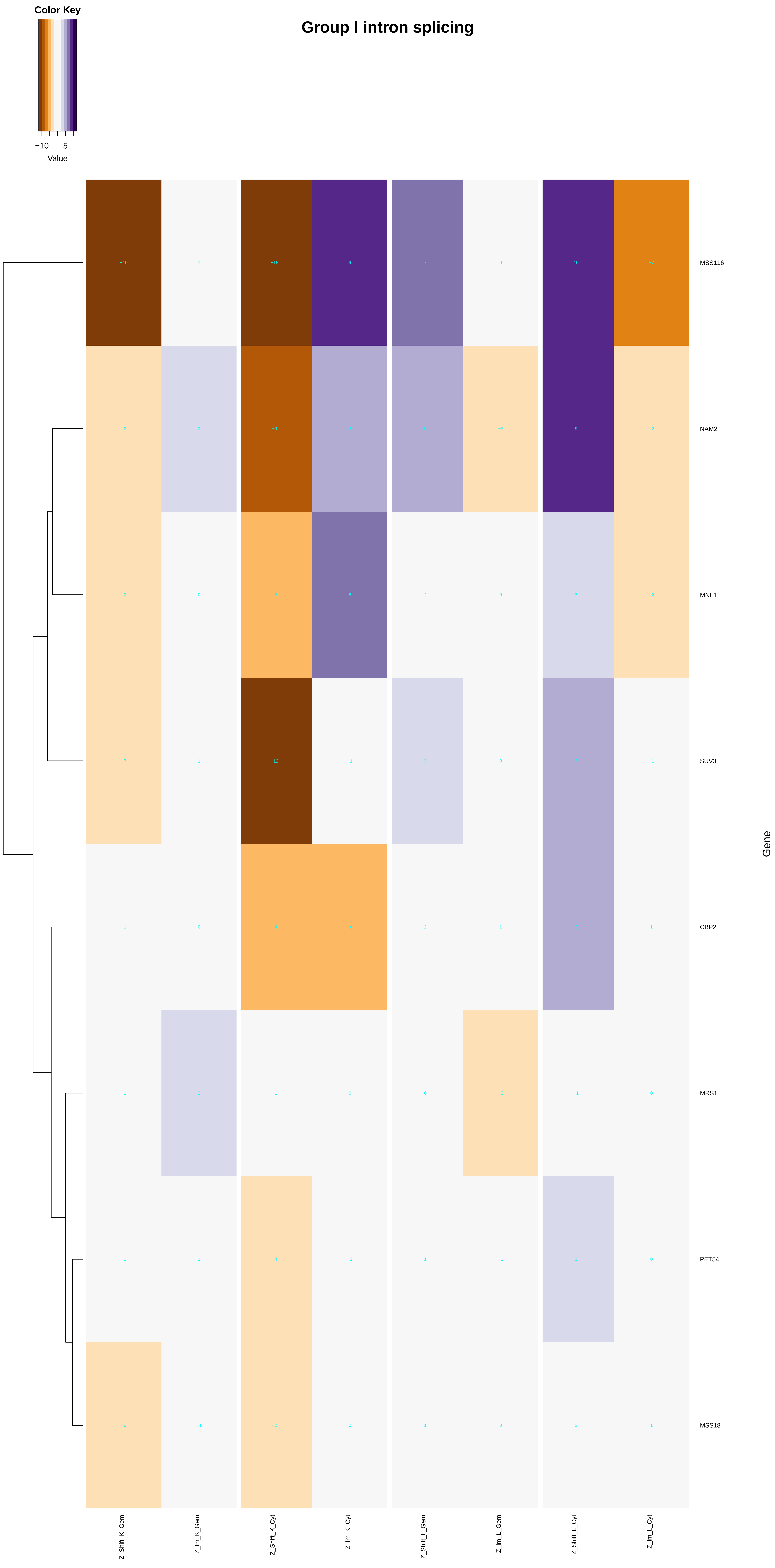

Color Key
−10
5
Value
Group I intron splicing
MSS116
NAM2
MNE1
SUV3
CBP2
MRS1
PET54
MSS18
Gene
Z_Shift_K_Gem
Z_Im_K_Gem
Z_Shift_K_Cyt
Z_Im_K_Cyt
Z_Shift_L_Gem
Z_Im_L_Gem
Z_Shift_L_Cyt
Z_Im_L_Cyt

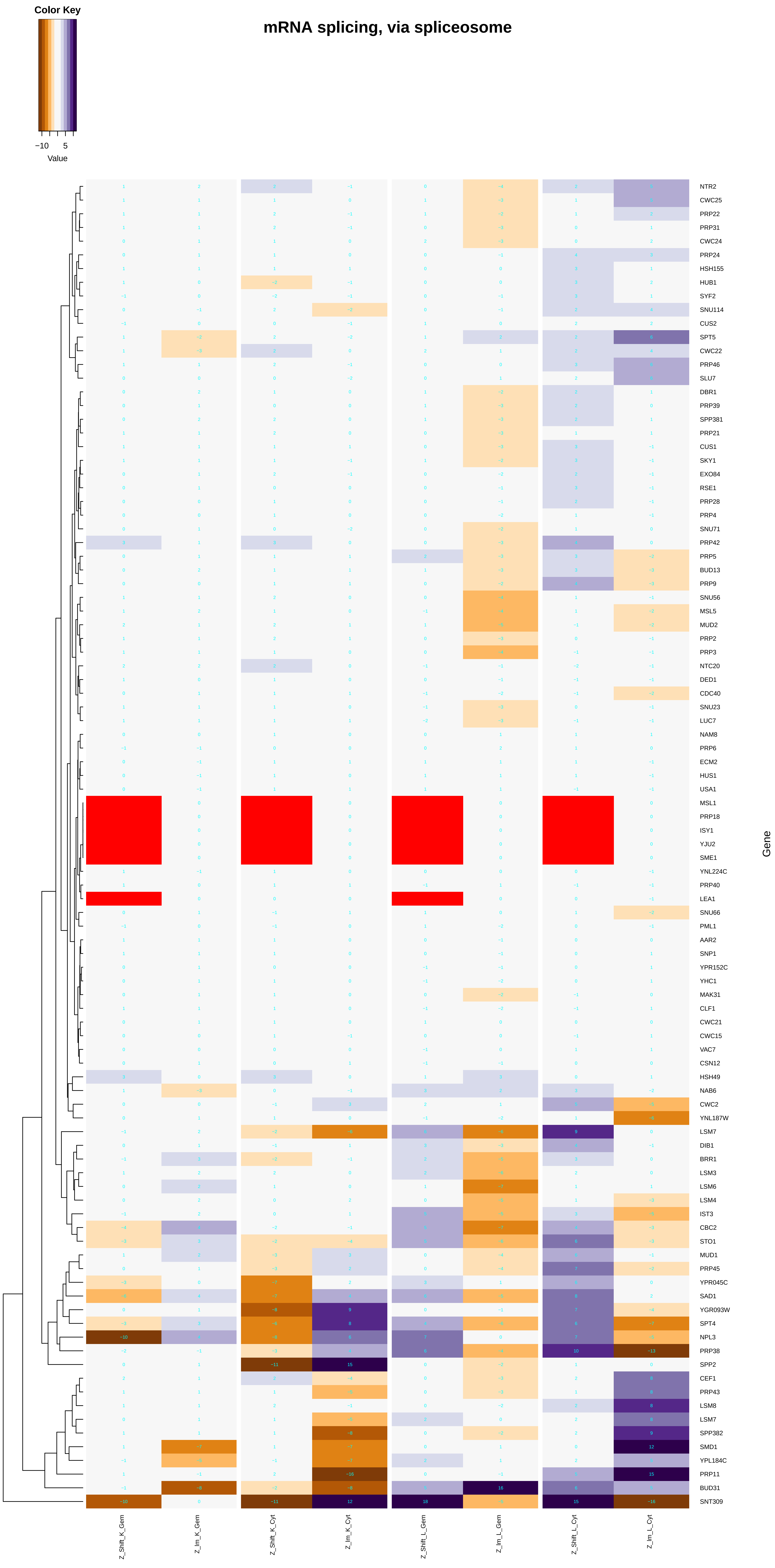
Color Key
−10
5
Value
mRNA splicing, via spliceosome
NTR2
CWC25
PRP22
PRP31
CWC24
PRP24
HSH155
HUB1
SYF2
SNU114
CUS2
SPT5
CWC22
PRP46
SLU7
DBR1
PRP39
SPP381
PRP21
CUS1
SKY1
EXO84
RSE1
PRP28
PRP4
SNU71
PRP42
PRP5
BUD13
PRP9
SNU56
MSL5
MUD2
PRP2
PRP3
NTC20
DED1
CDC40
SNU23
LUC7
NAM8
PRP6
ECM2
HUS1
USA1
MSL1
PRP18
ISY1
YJU2
SME1
YNL224C
PRP40
LEA1
SNU66
PML1
AAR2
SNP1
YPR152C
YHC1
MAK31
CLF1
CWC21
CWC15
VAC7
CSN12
HSH49
NAB6
CWC2
YNL187W
LSM7
DIB1
BRR1
LSM3
LSM6
LSM4
IST3
CBC2
STO1
MUD1
PRP45
YPR045C
SAD1
YGR093W
SPT4
NPL3
PRP38
SPP2
CEF1
PRP43
LSM8
LSM7
SPP382
SMD1
YPL184C
PRP11
BUD31
SNT309
Gene
Z_Shift_K_Gem
Z_Im_K_Gem
Z_Shift_K_Cyt
Z_Im_K_Cyt
Z_Shift_L_Gem
Z_Im_L_Gem
Z_Shift_L_Cyt
Z_Im_L_Cyt

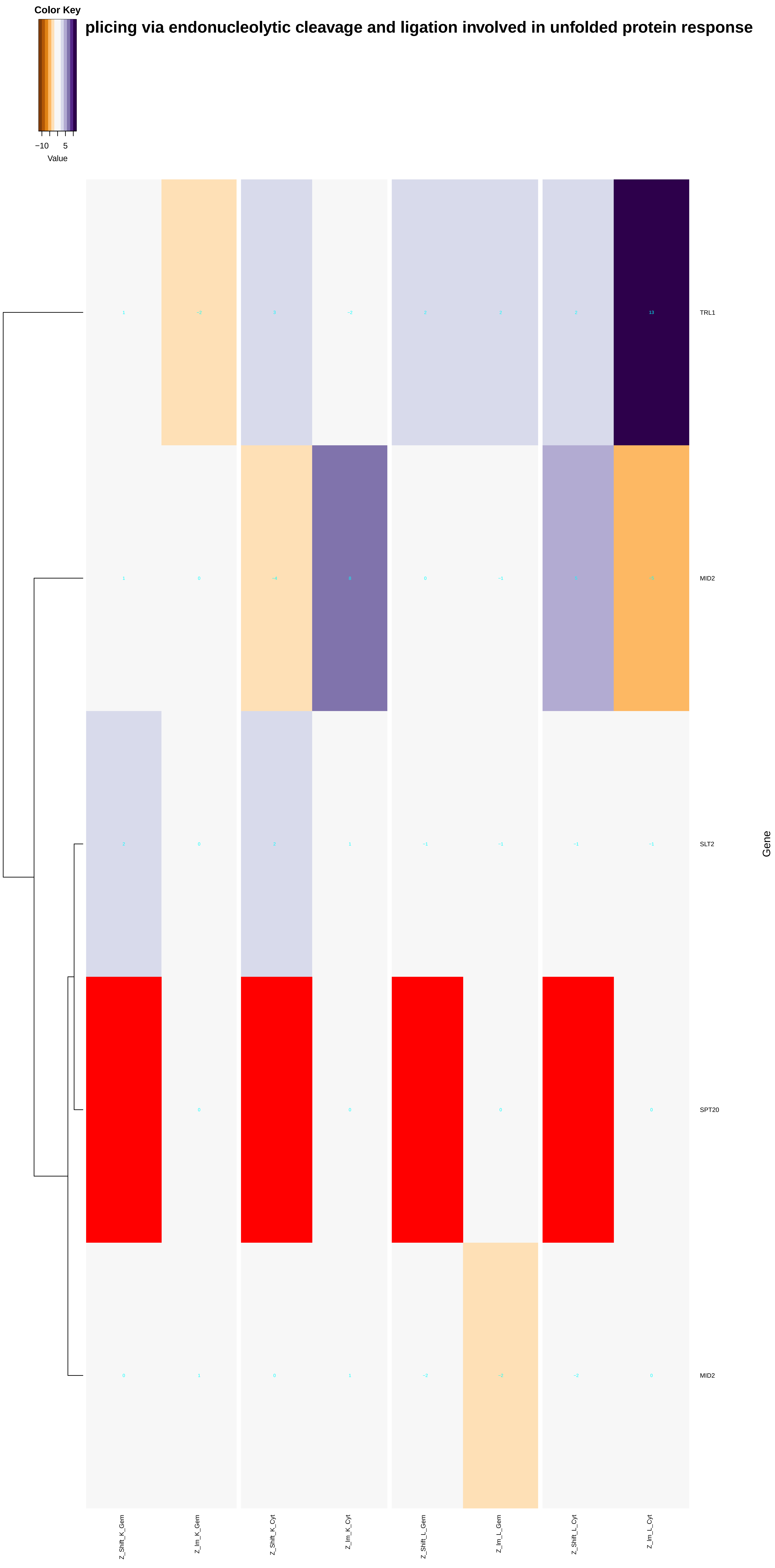
plicing via endonucleolytic cleavage and ligation involved in unfolded protein response
Color Key
−10
5
Value
TRL1
MID2
SLT2
SPT20
MID2
Gene
Z_Shift_K_Gem
Z_Im_K_Gem
Z_Shift_K_Cyt
Z_Im_K_Cyt
Z_Shift_L_Gem
Z_Im_L_Gem
Z_Shift_L_Cyt
Z_Im_L_Cyt

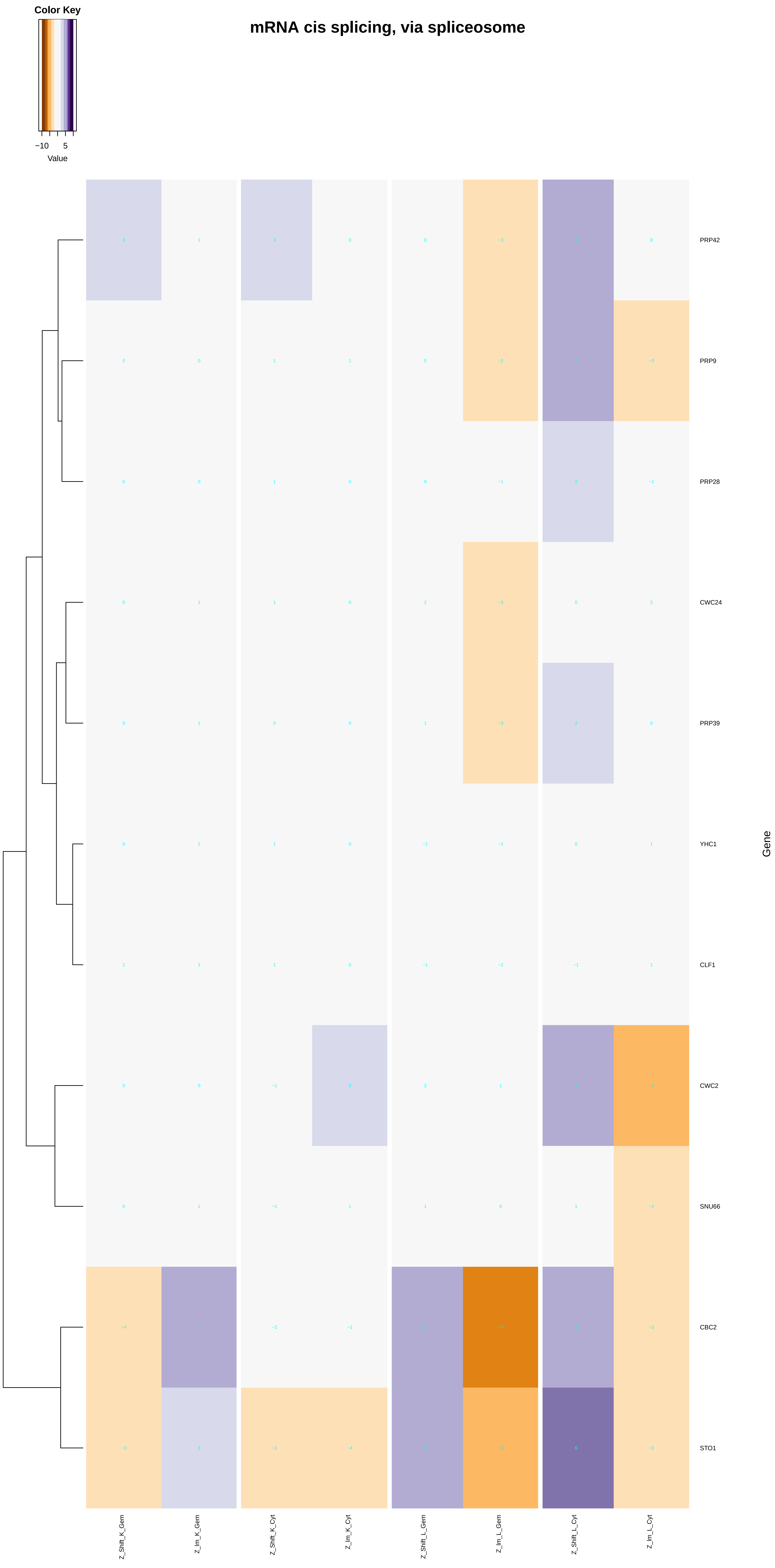
mRNA cis splicing, via spliceosome
Color Key
−10
5
Value
PRP42
PRP9
PRP28
CWC24
PRP39
YHC1
CLF1
CWC2
SNU66
CBC2
STO1
Gene
Z_Shift_K_Gem
Z_lm_K_Gem
Z_Shift_K_Cyt
Z_lm_K_Cyt
Z_Shift_L_Gem
Z_lm_L_Gem
Z_Shift_L_Cyt
Z_lm_L_Cyt
